# Appendiceal neoplasms and histological involvement of the mesoappendix: A case series

**DOI:** 10.1016/j.amsu.2020.05.037

**Published:** 2020-06-12

**Authors:** Jaideep Singh Rait, Joshua McGillicuddy, Jirayr Ajzajian

**Affiliations:** a William Harvey Hospital, East Kent NHS Trust, Kennington Rd, Willesborough, Ashford TN24 0LZ, United Kingdom; b Darent Valley Hospital, Dartford and Gravesham NHS Trust, Dartford, DA2 8DA, United Kingdom; c Medway Maritime Hospital, Medway NHS Trust, Windmill Road, Gillingham, Kent, ME7 5NY, United Kingdom

**Keywords:** Appendix, Appendectomy, Appendicectomy, Mesoappendix, Neuroendocrine

## Abstract

**Introduction:**

Acute appendicitis is a common presentation to surgical departments, typically resulting in appendicectomy. Appendiceal tumours may not be visible intraoperatively, and are present in roughly 0.5–1% of specimens. Routine resection of the mesoappendix is not universally practiced, despite the mesoappendix being commonly involved in appendiceal tumours.

This is a case series of the histological findings of 21 patients with appendiceal tumours, with consideration to tumour within the resected mesoappendix.

**Methods:**

We reviewed the histology of 1344 patients undergoing laparoscopic appendicectomy over a 6-year period assessing for the presence of appendiceal tumours and resected mesoappendix.

**Results:**

Twenty-one patients were found to have appendiceal tumours, with a mean maximum tumour dimension of 7.2 mm. Sixteen of these patients had simultaneous mesoappendix resection, of whom six (38%) were found to have direct or indirect tumour tissue within the mesoappendix.

**Conclusion:**

Further evidence for routine removal of the mesoappendix, and the need for larger prospective studies to investigate for any survival benefit. We note the worrying trend of conservative management of acute appendicitis.

## Highlights

• The mesoappendix is a key site of appendiceal tumour spread, directly & indirectly.
• Routine mesoappendix resection may reduce the need for reoperation.
• Conservative management of appendicitis may delay diagnosis of appendiceal tumours.

## 1 Introduction

Acute appendicitis is a common surgical condition requiring admission and emergency/urgent operation. It has a bimodal peak onset, in the third and seventh decades. The traditional presentation is that of periumbilical pain migrating to the right iliac fossa over McBurney's point as the inflammation involves the parietal peritoneum and localises. It is notorious for a myriad of presentations, often due to the variable positioning, meaning many uncertain appendicectomies are performed, with a UK negative appendicectomy rate of 20.6% [1], and US of 11.8% [2]. Scoring systems such as the Alvarado score are increasingly being used in an attempt to reduce this surgical burden of normal appendicectomies and inherent surgical risk. Tissue specimens from appendicectomy are routinely sent for histological analysis to assess for appendiceal neoplasms, found in around 0.5–1% of cases of acute appendicitis [3,4]. The typical appendiceal tumour is asymptomatic until appendiceal obstruction, neuroendocrine in origin, and picked up incidentally after resection.

Tumours of the appendix may arise from various cell types, including epithelial enterocytes, sub-epithelial neuroendocrine cells, and goblet cells. Neoplasms arising from enterocytes have a propensity to form mucin, and so can be classified as mucinous or non-mucinous [5]. Mucinous appendiceal neoplasms however have a unique biological behaviour and can prove difficult to characterise as either a benign adenoma or malignant adenocarcinoma [6]. Cancer of the appendiceal goblet cells is termed goblet cell carcinoma. These are rare, have a poor prognosis, and again are difficult to definitively classify due to their varied phenotype [7]. There is ongoing discussion by Tang et al., whether GCCs should be classified as ANETs or de novo mucous adenocarcinomas of the appendix [8].

Due to the complexity in classification of ANETs, various systems have been proposed, including the 2010 WHO classification [9] and the 2016 Modified Delphi Consensus [10]. The 2010 WHO system grades appendicular NETs as ‘*(1a) Well differentiated NETs with benign biological behaviour or (1b) Well differentiated NETs with uncertain malignant potential; (2) Well differentiated neuroendocrine carcinoma (with low malignant potential); and (3) Mixed exocrine-neuroendocrine carcinoma. Goblet cell carcinoma belongs to the last category’* [9,11].

As summarised in Table 1, there are consensus guidelines [12] on the management of appendiceal tumours, ranging from simple appendicectomy for tumours <1 cm, right hemicolectomy for features such as larger tumours, deep invasion, and positive surgical resection margins, to adding bilateral salpingo-oophorectomy and adjuvant intraperitoneal chemotherapy for peritoneally disseminated goblet cell carcinoma (pseudomyxoma peritonei). It is worth highlighting that during operations involving bowel resection, standard practice is to remove the accompanying mesentery, in part due to the spread of cancer along lymphatic supply following blood supply. This is not so for the appendix and its mesoappendix, even though mesoappendiceal involvement of appendiceal tumours can be shown in 10–20% of adults and 30–40% of children [[12], [13], [14], [15]].

**Table 1:** Recommended surgical strategies for appendiceal NETs based on specific clinical and histological characteristics. Reprinted from Griniatsos, Michail, WJGO 2010 [11].

Indications	Type of operation
Tumour size <1 cm	Appendicectomy
Tumour size 1–2 cm	Appendicectomy + Regular F/Up for 5 years
Tumour size >2 cm	Right hemicolectomy
Location of the tumour at the base of the appendix	Right hemicolectomy
Infiltration of the cecum	Right hemicolectomy
Positive surgical resection margins	Right hemicolectomy
Appendiceal mesentery invasion	Right hemicolectomy
Metastatically infiltrated mesoappendiceal lymph node	Right hemicolectomy
Presence of undifferentiated or low differentiated cells	Right hemicolectomy
Presence of goblet cells	
Goblet cell carcinoma in males	Right hemicolectomy
Goblet cell carcinoma in females (regardless of age)	Right hemicolectomy + Bilateral salpingo-oophorectomy
Peritoneal dissemination from goblet cell carcinoma	Cytoreductive surgery + Adjuvant intraperitoneal chemotherapy

Due to differences in training and inclination, there is heterogeneity between surgeons regarding appendicectomy technique, either practicing on bloc appendiceal and mesoappendiceal resection, or skeletisation and sole removal of the appendix. There is currently insufficient evidence as to whether routine removal of the mesoappendix affects spread of appendiceal neuroendocrine tumour, survival rate, or has an increased rate of complications.

## 2 Methods

No trial registration was undertaken and all patient data was anonymised.

This case series has been reported according to the PROCESS guidelines [16].

This is a retrospective, single-centre, consecutive case series of all patients over the age of five who underwent laparoscopic appendicectomy for sufficient clinical suspicion of acute appendicitis over a six-year period (2012–2018) in a community district general hospital (DGH) in the South East of England. We did not include open appendicectomies, giving the same exclusion criteria as laparoscopic contraindications – here being <35 kg, due to the difficulty of laparoscopic technique in too small an abdomen.

Resected appendiceal tissue was routinely sent for histological analysis. For all patients identified to have any neoplasm of the appendix, we collected age, gender, use of pre-operative imaging and the radiological finding, length of stay, whether the mesoappendix was resected and the full histopathological result.

The laparoscopic appendicectomies were performed by general surgical consultants and registrars (speciality training years 3–8), with or without direct consultant supervision. Histology reporting was performed or reviewed by at least one consultant histopathologist to ensure reliable and accurate reporting.

Patients in the UK who are found to have tumours of the appendix are discussed at a local multi-disciplinary team (MDT) meeting consisting of consultants in pathology, radiology, surgery and oncology, and are then referred to specialist local referring centres. Patients were followed up to their MDT outcomes but not eventual clinical outcome.

## 3 Results

Patient characteristics are outlined in Table 2. Mean age was 41, with a wide range from 19 to 71 years, and there was a female preponderance of cases (71%).

**Table 2:** Patient characteristics.

Characteristics	Sub-characteristics	Number of patients (%)
**Sex**	Male Female	6 (29) 15 (71)
**Age in years**	Mean Range	41 19–71
**Pre-operative imaging**	Ultrasound CT No Imaging	11 (52) 5 (24) 6 (29)
**Further operation required**	Yes No	5 (24) 16 (76)
**Length of stay**	Mean Range	3.1 days 1–6 days
**Tumour size (maximum dimension)**	Mean Range	7.2 mm 1.5–20 mm
**Tumour type**	Well differentiated neuroendocrine Goblet-cell Mucinous Mixed carcinoid-adenocarcinoma	16 (76) 2 (10) 2 (10) 1 (5)

During the six-year study period, 1344 patients underwent laparoscopic appendicectomy. Twenty-one patients were found to have tumours of the appendix. Tumour size ranged from a maximum dimension of 1.5 mm–20 mm with a mean of 7.2 mm. There were sixteen neuroendocrine tumours and five epithelial tumours, which can be sub-categorised into one mixed carcinoid-adenocarcinoma, two mucinous and two goblet-cell tumours. There were none of the rarer primary appendiceal tumour subtypes, including lymphomas, mesenchymal tumours and sarcomas.

Five patients had a preoperative CT, eleven had a preoperative ultrasound scan, and only seven uses of imaging were suggestive of appendicitis, with none suggesting appendiceal neoplasms.

As outlined in Table 3, the mesoappendix was identified in the tissue resections of sixteen out of the twenty-one patients. Of these, six specimens (38%) had tumour tissue within the mesoappendix – four directly from an appendiceal locus and two indirectly. We make special note of these latter two patients. Firstly, a goblet-cell carcinoma that appears to not directly grow into the mesoappendix but is nevertheless present within the mesoappendix by means of perineural/intraneural invasion, and the second was thought to be a multifocal synchronous appendiceal tumour.

**Table 3:** Resection and tumour invasion of the mesoappendix.

	Mesoappendix resected	Mesoappendix not resected
Tumour invasion into mesoappendix present	6	–
Tumour invasion into mesoappendix not identified	10	5

Based on recommended ANET surgical strategy from the consensus guidelines, five patients were indicated for a right hemicolectomy, with one including bilateral salpingo-oophorectomy.

## 4 Discussion

The concerning prevalence of tumour invading the mesoappendix both directly and indirectly, macroscopically and microscopically, highlights the need for routine complete mesoappendiceal resection. Ours is not a novel proposition; In 2013, Davenport et al. recommended routine ‘en bloc mesoappendix resection during appendicectomy’ [17]. Doing so is generally very safe compared to solely removing the appendix, but possible complications could include more difficult haemostasis and failure to remove the entire mesoappendix.

Routine removal of the mesoappendix would likely provide a higher proportion of clear resection margins and therefore fewer reoperations, alongside more reliable staging and grading of appendiceal tumours.

Our study corroborates other studies the incidence of NEATs is around 1%, with a female preponderance of cases [12], although we do not know the gender distribution who underwent laparoscopic appendicectomy in our department.

We found pre-operative imaging was not useful with regards to appendiceal tumour identification. There have been advances in imaging in this regard [18], but given the low pre-test probability and the small size of the tumour, this is of uncertain use at present.

Conservative management of appendicitis may delay diagnosis of appendiceal tumours in a significant proportion of patients with tumour related acute appendicitis, especially given the poor quality of preoperative imaging to identify both appendicitis and appendiceal tumours. As appendiceal tumours are often picked up incidentally after resection, if a tumour-related appendicitis temporarily resolves with conservative management, there may be cancerous spread by the time the tumour causes a second presentation.

### 4.1 Limitations and relevance of the study

This was a study with a reasonable sample size and range of tumours, with resected tissue undergoing a thorough and robust histological analysis.

One limitation is that we have not followed up patients to clinical outcomes, so cannot comment on exactly how many reoperations were performed and the indication, or any subsequent findings of non-direct tumour invasion.

We recommend further studies with regard to both short- and long-term outcomes of routine removal of the mesoappendix during appendicectomy, specifically assessing possible complications of removal, and the risks of inadequate tumour resection.

Appendicectomies are performed all around the globe and evidence as to whether routine removal of the mesoappendix affects patient outcomes could have significant impact on surgical management of appendicitis.

## 5 Conclusions

This case series provides further justification for routine removal of the mesoappendix, and its consideration in future surgical guidelines, given the mesoappendix is a key area of appendiceal tumour spread.

It also highlights the potentially concerning trend of conservative management of acute appendicitis, and the need for more data with regards to possible subsequent delays in diagnoses of appendiceal tumours.

## Ethical approval

None declared.

## Sources of funding

None declared.

## Consent

Consent was not possible retrospectively as all patient data was anonymised.

## Declaration of conflicting interestCOI

None declared.

## Provenance and peer review

Not commissioned, externally peer reviewed.

## Author contribution

Jaideep Singh Rait: Study concept, data collection & interpretation, writing the paper. Joshua McGillicuddy: Study direction, data interpretation and writing the paper. Jirayr Ajzajian: Data interpretation and writing the paper.

## Guarantor

Jaideep Singh Rait accept full responsibility for the work and conduct of the study, have had access to the data and controlled the decision to publish.
